# Identification and characterization of heat-responsive lncRNAs in maize inbred line CM1

**DOI:** 10.1186/s12864-022-08448-1

**Published:** 2022-03-16

**Authors:** Xiaolin Hu, Qiye Wei, Hongying Wu, Yuanxiang Huang, Xiaojian Peng, Guomin Han, Qing Ma, Yang Zhao

**Affiliations:** The National Engineering Laboratory of Crop Stress Resistance Breeding, School of Life Sciences, Anhui Agricultural University, Hefei, 203036 China

**Keywords:** Maize, Heat stress, LncRNA, Target genes, Regulatory network

## Abstract

**Background:**

Frequent occurrence of extreme high temperature is a major threat to crop production. Increasing evidence demonstrates that long non-coding RNAs (lncRNAs) have important biological functions in the regulation of the response to heat stress. However, the regulatory mechanism of lncRNAs involved in heat response requires further exploration and the regulatory network remains poorly understood in maize.

**Results:**

In this research, high-throughput sequencing was adopted to systematically identify lncRNAs in maize inbred line CM1. In total, 53,249 lncRNAs (259 known lncRNAs and 52,990 novel lncRNAs) were detected, of which 993 lncRNAs showed significantly differential expression (DElncRNAs) under heat stress. By predicting the target genes, 953 common targets shared by *cis*- and *trans*-regulation of the DElncRNAs were identified, which exhibited differential expression between the control and the heat stress treatments. Functional annotation indicated that a number of important biological processes and pathways, including photosynthesis, metabolism, translation, stress response, hormone signal transduction, and spliceosome, were enriched for the common targets, suggesting that they play important roles in heat response. A lncRNA-mediated regulatory network was constructed to visualize the molecular response mechanism in response to heat stress, which represented the direct regulatory relationships of DElncRNAs, differentially expressed miRNAs, target genes, and functional annotations.

**Conclusions:**

This study lays a foundation for further elucidation of the regulatory mechanism for the response to heat stress in the maize inbred line CM1. The findings provide important information for identification of heat-responsive genes, which will be beneficial for the molecular breeding in the cultivation of heat-tolerant maize germplasm.

**Supplementary Information:**

The online version contains supplementary material available at 10.1186/s12864-022-08448-1.

## Background

More than 90% of the eukaryotic genome can be transcribed into RNAs of which non-coding RNAs account for most of these transcripts [1, 2]. On the basis of their length, non-coding RNAs can be categorized into small RNAs (< 40 nt) and long non-coding RNAs (lncRNAs, > 200 nt) [3–5]. Considerable progress has been achieved in understanding the functions and regulatory mechanisms of microRNAs (miRNAs), which comprise an important type of small RNAs. However, the biological functions of lncRNAs remain largely unknown [6]. LncRNAs exhibit a low degree of conservation in sequence length, varying from 200 to tens of thousands of nucleotides [7]. Similar to classical mRNAs, lncRNAs are mainly transcribed by RNA polymerase II in eukaryotes, but often exhibit a lower expression level in a tissue-specific or cell-type-specific expression pattern without an open reading frame [8, 9]. On the basis of their chromosomal location and orientation, lncRNAs can be categorized into sense lncRNAs, antisense lncRNAs, intronic lncRNAs and intergenic lncRNAs (lincRNAs) [1, 10].

Using high-throughput sequencing, systematic identification of lncRNAs in many plant species has been conducted in recent years. For example, 6,480 transcripts have been defined as lincRNAs in Arabidopsis based on an integrative analysis of 200 transcriptome data sets [11]. In rice, 1,624 lincRNAs and 600 long non-coding natural antisense transcripts, were identified using multiple RNA-sequencing (RNA-seq) datasets [12]. By integrating multiple expression and RNA-seq datasets, a total of 20,163 lncRNA candidates were identified in maize of which 1,704 were considered to be high-confidence lncRNAs [10]. Increasing evidence indicates that lncRNAs regulate gene expression through a variety of mechanisms in diverse developmental processes, such as plant flowering, sexual reproduction, photomorphogenesis, and male sterility [12–15]. The main regulatory mechanisms by which plant lncRNAs regulate gene expression involve mediating chromatin remodeling, and acting as precursors of miRNAs and as natural antisense transcripts of mRNAs [16, 17]. For example, Arabidopsis cold-assisted intronic noncoding RNA (*COLDAIR*) and cold-induced long antisense intragenic RNA (*COOLAIR*) are involved in the regulation of flowering by participating in epigenetic silencing of *FLOWERING LOCUS C* (*FLC*), which functions as a regulator of vernalization-controlled flowering [13, 18].

Environmental factors, such as high temperature and drought, can severely impact on plant growth and development. Expression of a series of functional proteins and regulatory factors is induced during adaptation to abiotic stress. LncRNAs also participate in the regulation of abiotic stress responses and a large number of detected lncRNAs exhibit significant changes in expression under stress treatments [19–22]. Importantly, the function of several plant lncRNAs in abiotic stress responses have been characterized. For example, *npc536* is a salt-responsive lncRNA identified in Arabidopsis. The expression of *npc536* is up-regulated in roots and leaves under salt treatment, and overexpression of *npc536* enhances root growth under salt stress in Arabidopsis [23]. In rice, the lncRNA *TCONS_00021861* functions as an important regulator associated with drought tolerance and regulates the expression of an indole-3-pyruvate monooxygenase gene, *YUCCA7*, through interaction with miR528-3p [24]. In wheat, three lncRNAs, comprising lncR9A, lncR117, and lncR616, interact with miR398; overexpression of these lncRNAs in transgenic plants increases tolerance to cold stress by competitively binding to miR398 to regulate expression of *COPPER/ZINC SUPEROXIDE DISMUTASE 1* (*CSD1*) [25]. In cotton, *lncRNA973* expression is up-regulated under salt treatment and *lncRNA973*-overexpression transgenic plants exhibit increased tolerance to salt stress [26]. Therefore, identification of stress-responsive lncRNAs is of great importance for molecular breeding for plant stress tolerance.

Heat stress is among the most prevalent environmental factors that affect maize growth and development. In particular, the frequent occurrence of high temperature in the Huang-huai-hai region of China has severely affected maize yields and quality in recent years. In our previous study, we performed a comparative phenotype and transcriptome analysis of the maize hybrid An’nong 591 and its parental lines (paternal line CM1 and maternal line CB25) under control and heat stress treatments. The elite inbred line CM1 shows strong resistance to heat stress and may contributed the genes conferring heat resistance in An’nong 591 [27]. As already mentioned, lncRNAs are involved in many biological processes and abiotic stress responses. However, their functions and molecular mechanisms in response to heat stress remain poorly understood in maize. In the current work, high-throughput sequencing was adopted to identify lncRNAs involved in the heat response in seedlings of the heat-resistant maize inbred line CM1. A systematic analysis of the sequence structure, expression profile, and target prediction for the detected lncRNAs was performed. Importantly, a lncRNA-mediated regulatory network for the heat stress response was constructed using Cytoscape software. The present results provided an important foundation for further functional research and molecular breeding for heat stress resistance in maize.

## Materials and methods

### Plant material, RNA extraction and cDNA library construction

To identify lncRNAs responsive to heat stress, a heat-resistant elite maize inbred line, CM1, was selected for high-throughput sequencing. Seedlings of CM1 were grown in a plant light incubator at 28 °C/23 °C (day/night) under a photoperiod of 16 h/8 h (light/dark) as the non-stress condition (control). For heat stress treatment, seedlings at the three-leaf stage were treated with 42 °C for 24 h. Twenty seedlings were grown for each treatment. Leaves were sampled from seedlings of uniform growth for each treatment. All leaf samples were immediately frozen in liquid nitrogen and used for high-throughput sequencing. Sampling from each group comprised three biological replicates (control group: PC1, PC2 and PC3; heat treatment group: PH1, PH2 and PH3). Extraction and quality evaluation of total RNAs from the six samples were conducted in accordance with our previous study [28]. Ribosomal RNA was removed from the extracted RNA using the Ribo-Zero™ rRNA Removal Kit (Epicentre, USA). A cDNA library of each sample was constructed using the NEBNext® Ultra™ Directional RNA Library Prep Kit for Illumina® (NEB, USA), which included mRNA fragmentation, cDNA synthesis, adaptor ligation, PCR amplification, purification, and other crucial steps.

### Sequencing and bioinformatic identification of lncRNAs

The cDNA library quality was analyzed using an Agilent 2100 Bioanalyzer (Agilent Technologies, USA). After quality assessment, the constructed library was sequenced using a Novaseq 6000 platform. Paired-end reads of 2 × 150 bp were generated. To obtain clean reads, the raw reads were filtered to remove low-quality reads below the Q20 and Q30 thresholds, adaptor sequences, and reads containing poly-N. The clean reads were aligned to the maize reference genome (AGPv4) with HISAT (v2.0.4) [29]. StringTie (v1.3.3) was used to assemble the mapped reads into transcripts, and the number of fragments per kilobase per million mapped reads (FPKM) was calculated to determine the expression level of the assembled transcripts [30]. To identify lncRNA candidates from the assembled transcripts, the following main steps were adopted. First, transcripts ≥ 200 nt in length were selected for subsequent analysis and single-exon transcripts were retained for lncRNA screening. Second, transcripts that overlapped with known protein-coding genes were filtered out, and transcripts that overlapped with annotated lncRNAs in the database were retained for further analysis. Finally, transcripts with FPKM ≥ 0.5 were considered to be reliably expressed sequences. The protein-coding potential of all retained transcripts was determined using the Coding Potential Calculator (CPC), Coding-Non-Coding-Index (CNCI) and Pfam tools. Transcripts with protein-coding potential not shared by the three programs were considered to be novel lncRNAs [31–33].

### Differential expression analysis and qRT-PCR validation

The calculated FPKM values were used to determine the genes that were differentially expressed between the control and heat treatment using edgeR software [34]. Differentially expressed genes were determined with the criteria padj ≤ 0.05 and |log_2_foldchange|≥ 1. To validate the RNA-seq results, 10 differentially expressed lncRNAs (DElncRNAs) were selected for quantitative real-time PCR (qRT-PCR) analysis. Primers of the chosen lncRNAs were designed using Primer3Plus online software and are listed in Table S1. First-strand cDNA for each sample was synthetized from 1 μg RNA using the mix of oligo (dT) and random primers provided with the Evo M-MLV RT Mix Kit with gDNA Clean for qPCR (AG, China). The RT-PCR reactions were conducted on a Roche Light Cycler 480 II real-time PCR system (Roche, Germany) using FastStart Universal SYBR Green Master (Roche, Germany) with three technical and three biological replicates for each gene. The *GAPDH* gene (NM_001111943.1) was used as the endogenous control, and PCR procedure was conducted in accordance with our previous study [27], and the relative expression level was calculated using the 2^−ΔΔCt^ method [35].

### Prediction of target genes of DElncRNAs

To predict the possible biological functions of the lncRNAs, the *cis*- and *trans*-regulated target genes of the detected DElncRNAs were identified. Based on the genomic location, protein-coding genes located in the regions 100 kb upstream and downstream of the lncRNA were defined as the *cis*-target genes of lncRNAs. To identify the *trans*-target genes of lncRNAs, Pearson’s correlation coefficients (*R*) between lncRNAs and mRNAs were calculated on the basis of their expression profile. *Trans*-regulated target genes were predicted with an absolute *R* value ≥ 0.95. Differential expression analysis was conducted to identify important target genes involved in the heat stress response, and the target genes in common to *cis*- and *trans*-regulated members that were differentially expressed between the control and heat treatments were selected for subsequent analysis.

### GO and KEGG enrichment analysis

Gene Ontology (GO) enrichment analysis for the differentially expressed genes (target genes) was conducted using the GOseq R package [36]. The enrichment results were categorized into the biological process (BP), cell composition (CC), and molecular function (MF) categories. A pathway enrichment analysis was performed using the Kyoto Encyclopedia of Genes and Genomes (KEGG) database [37].

### Construction of lncRNA-mediated regulatory network for heat stress response

To construct a regulatory network in the response to heat stress in the maize inbred line CM1, the GO and KEGG annotations for the common target genes shared by *cis*- and *trans*-acting lncRNAs were used to explore the potential biological functions of DElncRNAs. In addition, 35 significantly differentially expressed miRNAs (DEMs) identified in our previous study [38], were used to predict the regulatory relationships between DEMs and DElncRNAs using psRNATarget software [39]. On the basis of these results, Cytoscape software was used to construct a lncRNA-mediated regulatory network to visualize the regulatory relationships of DElncRNAs, DEMs, target genes, and the significantly enriched GO terms and KEGG pathways (*p*-value < 0.05).

## Results

### Identification of lncRNAs by high-throughput sequencing

**Fig. 1:**
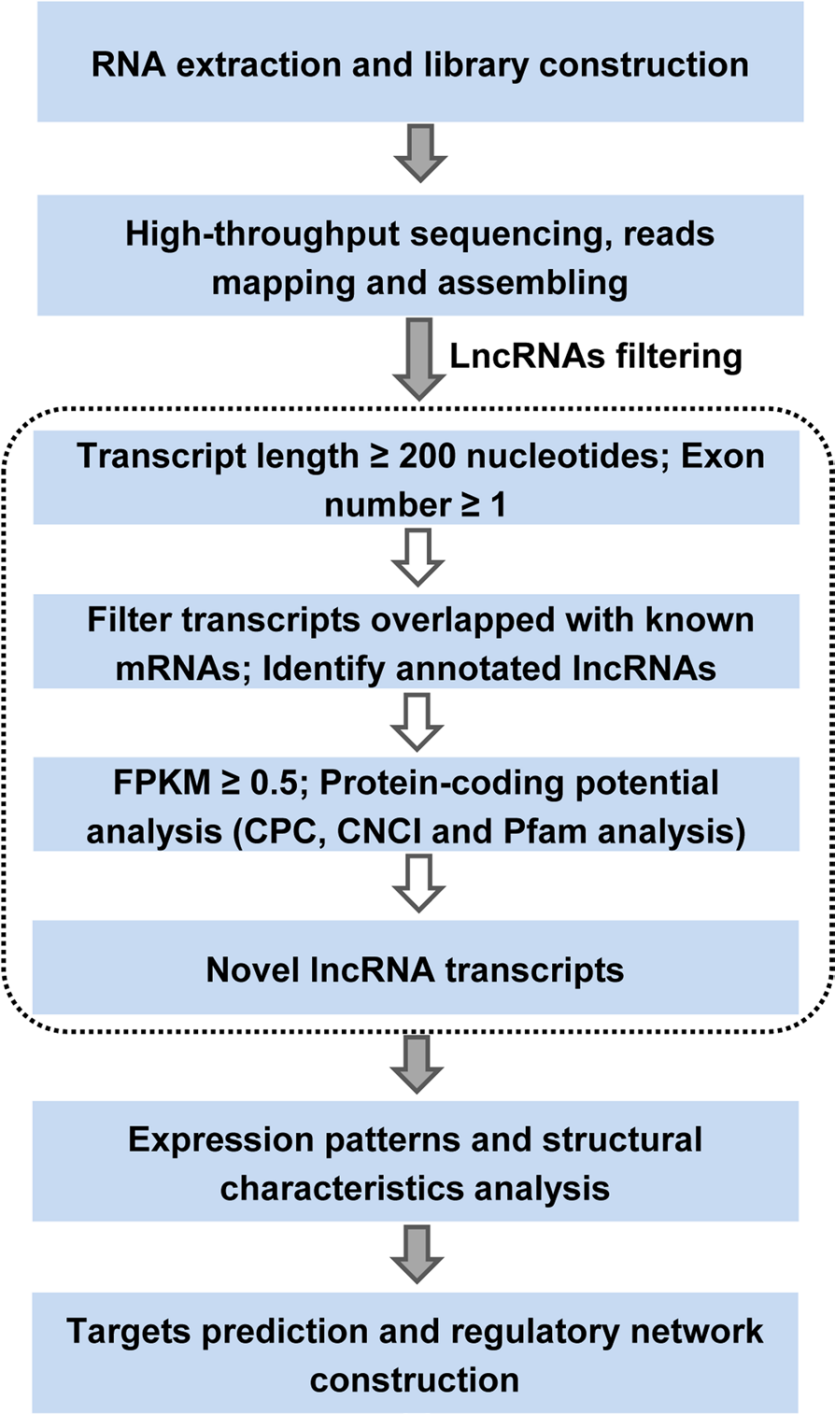
Flow diagram of procedure for identification of lncRNAs in maize

**Fig. 2:**
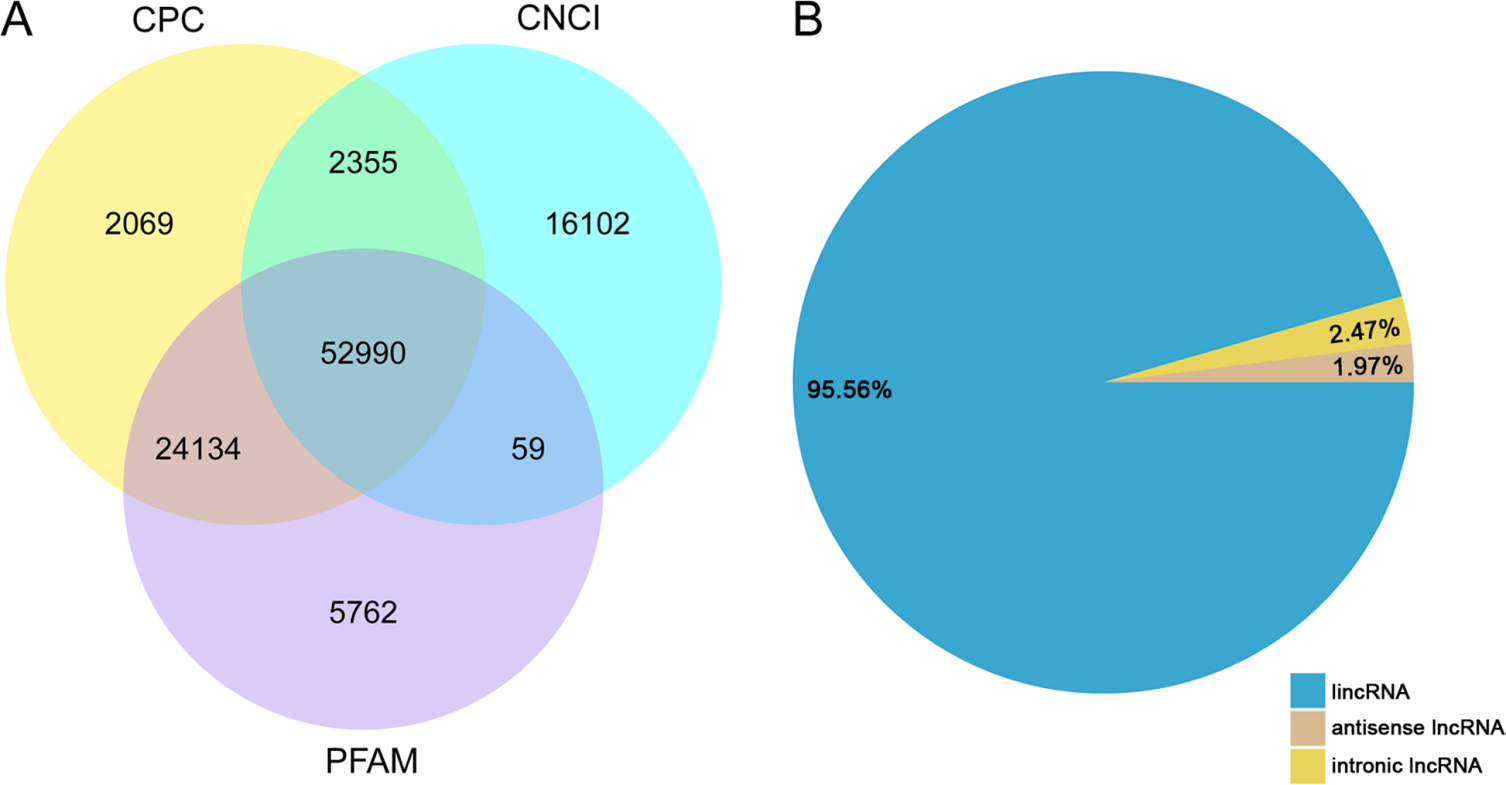
Coding potential prediction and classification of detected lncRNAs. **A** Protein-coding potential of lncRNAs predicted with the Coding Potential Calculator (CPC), Coding-Non-Coding Index (CNCI), and Pfam tools; **B** Classification of identified lncRNAs

To identify heat-responsive lncRNAs in the maize inbred line CM1, six lncRNA libraries were constructed from six samples of the control group (PC1-PC3) and heat treatment group (PH1-PH3), and used for subsequent high-throughput sequencing. After quality control of the raw data, 685,374,190 clean reads were generated from the six libraries; the total percentage of reads mapped to the maize reference genome for each library was not less than 81.17% (Table S2). The Q20 scores ranged from 96.81% to 97.22%, and the Q30 score of each library was greater than 91.22%. A total of 525,245 transcripts were assembled from the mapped reads from the six libraries. To identify lncRNA candidates, a bioinformatics pipeline was adopted to filter the assembled transcripts, as shown in the flow chart (Fig. 1). As a result, 52,990 transcripts were determined as reliably expressed novel lncRNAs without protein-coding potential based on the analysis with the CPC, CNCI, and Pfam tools (Fig. 2A). In addition, 259 known lncRNAs were identified that could be annotated in the database. The 53,249 lncRNAs were mainly divided into three types: lincRNAs accounted for 95.56% of the total lncRNAs, and the remainder were classified as intronic lncRNA (2.47%) or antisense lncRNA (1.97%) (Fig. 2B).

### Comparison of expression and structural characteristics of lncRNAs and mRNAs

**Fig. 3:**
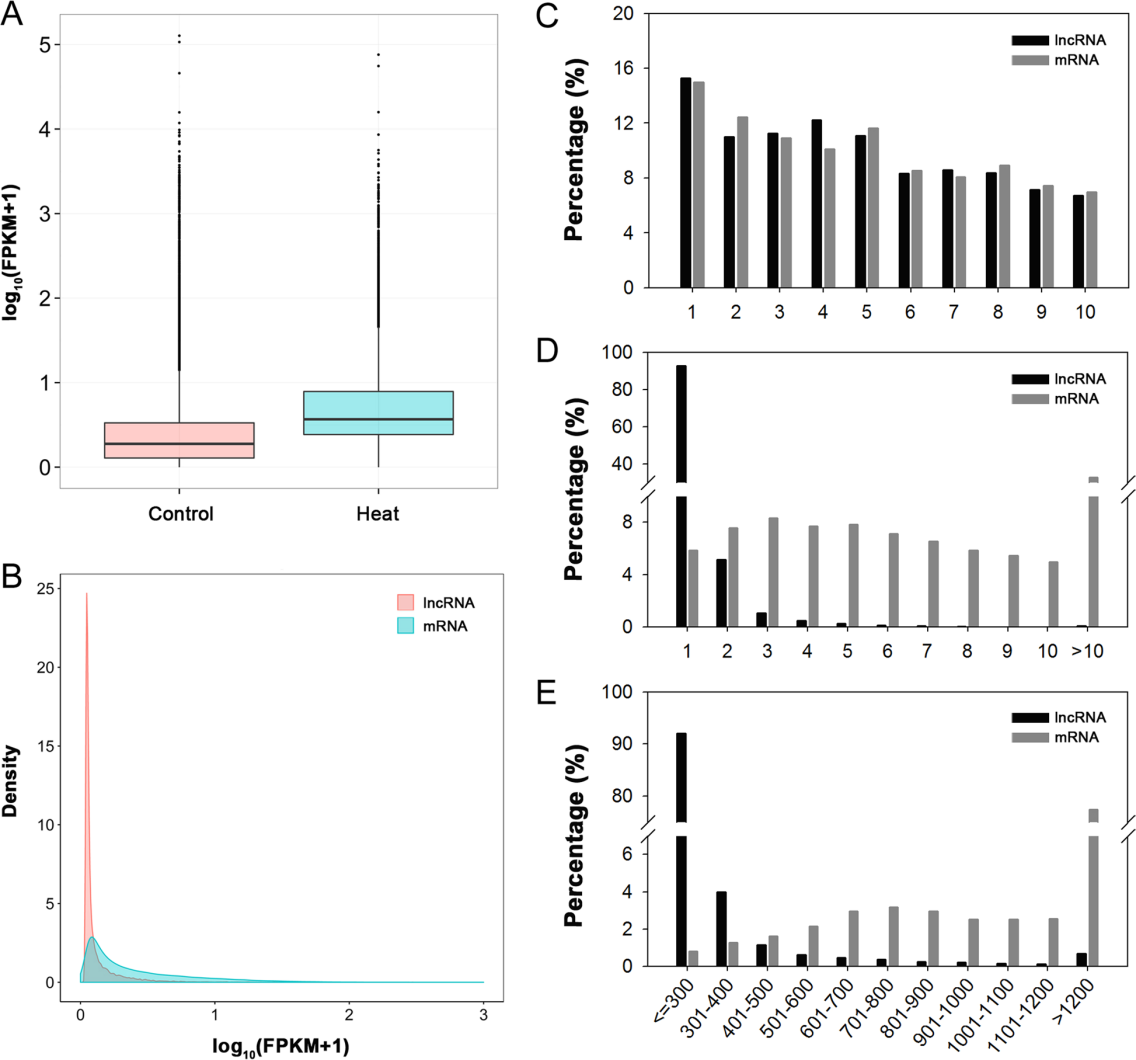
Comparative analysis of expression and structural characteristics between lncRNAs and mRNAs. **A** Expression level of transcripts under control and heat stress treatments; **B** Expression levels of lncRNAs and mRNAs; **C** Distribution of lncRNAs and mRNAs on maize chromosomes; **D** Exon number of lncRNAs and mRNAs; **E** Transcript length of lncRNAs and mRNAs

**Fig. 4:**
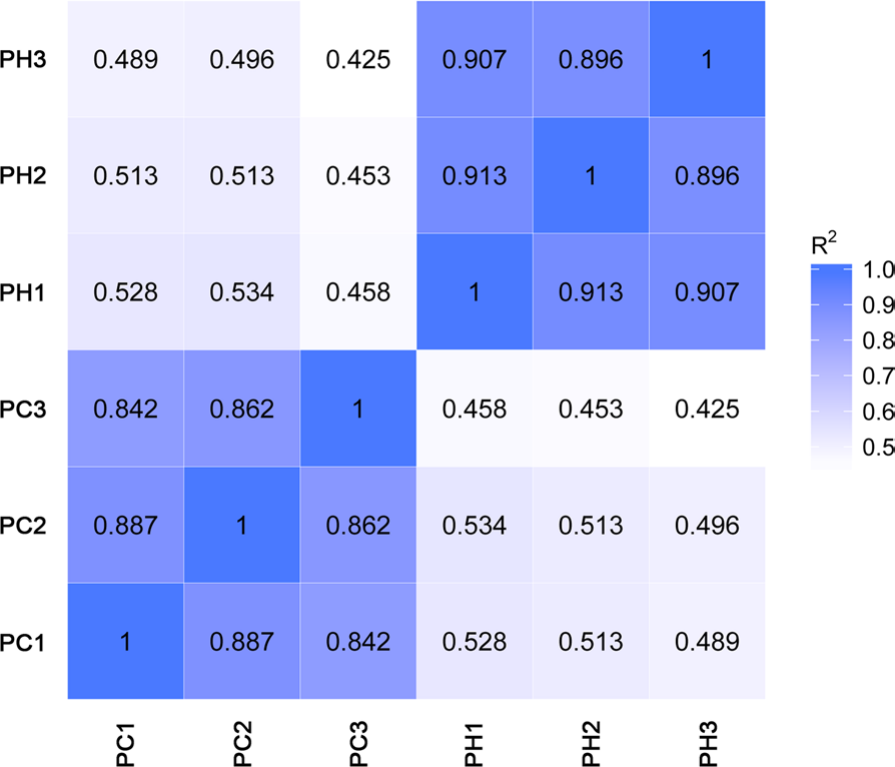
Correlation analysis of the six samples of the control and heat stress treatments. PC, seedlings grown under the control conditions; PH, seedlings subjected to heat stress treatment; 1–3, three biological replicates

Changes in expression of the retained transcripts in the control and heat stress treatments were investigated. The overall expression level of the transcripts (lncRNAs and mRNAs) under the heat stress treatment was significantly higher than that under non-stress condition, suggesting that the expression of many transcripts may be induced by heat stress (Fig. 3A). A comparative analysis of the expression profiles of lncRNAs and mRNAs revealed that the expression levels of lncRNAs were significantly lower than those of mRNAs, which was consistent with the findings of previous studies (Fig. 3B). We also performed a comparative analysis to explore differences in structural characteristics between lncRNAs and mRNAs. The results indicated that no significant difference was detected in the genomic locations of lncRNAs and mRNAs on the 10 maize chromosomes (Fig. 3C). However, significant differences in exon number and transcript length were observed. Among the detected lncRNAs, 92.64% contained only one exon, 5.16% contained two exons. In contrast, only 5.87% of mRNAs contained one exon, whereas 32.68% contained more than 10 exons (Fig. 3D). Transcript length of the detected lncRNAs ranged from about 300 to 1200 nt, and most lncRNAa were less than 500 nt in length (97.14%), whereas 77.48% of mRNAs were greater than 1200 nt in length (Fig. 3E). Correlation analysis of the six samples indicated that the correlation value (*R*^*2*^) of three replicates in the control group was greater than 0.842, whereas the value for the heat treatment group was greater than 0.896 (Fig. 4), suggesting that the repeatability of the biological replicates of each group was acceptable.

### Identification of heat-responsive lncRNAs

**Fig. 5:**
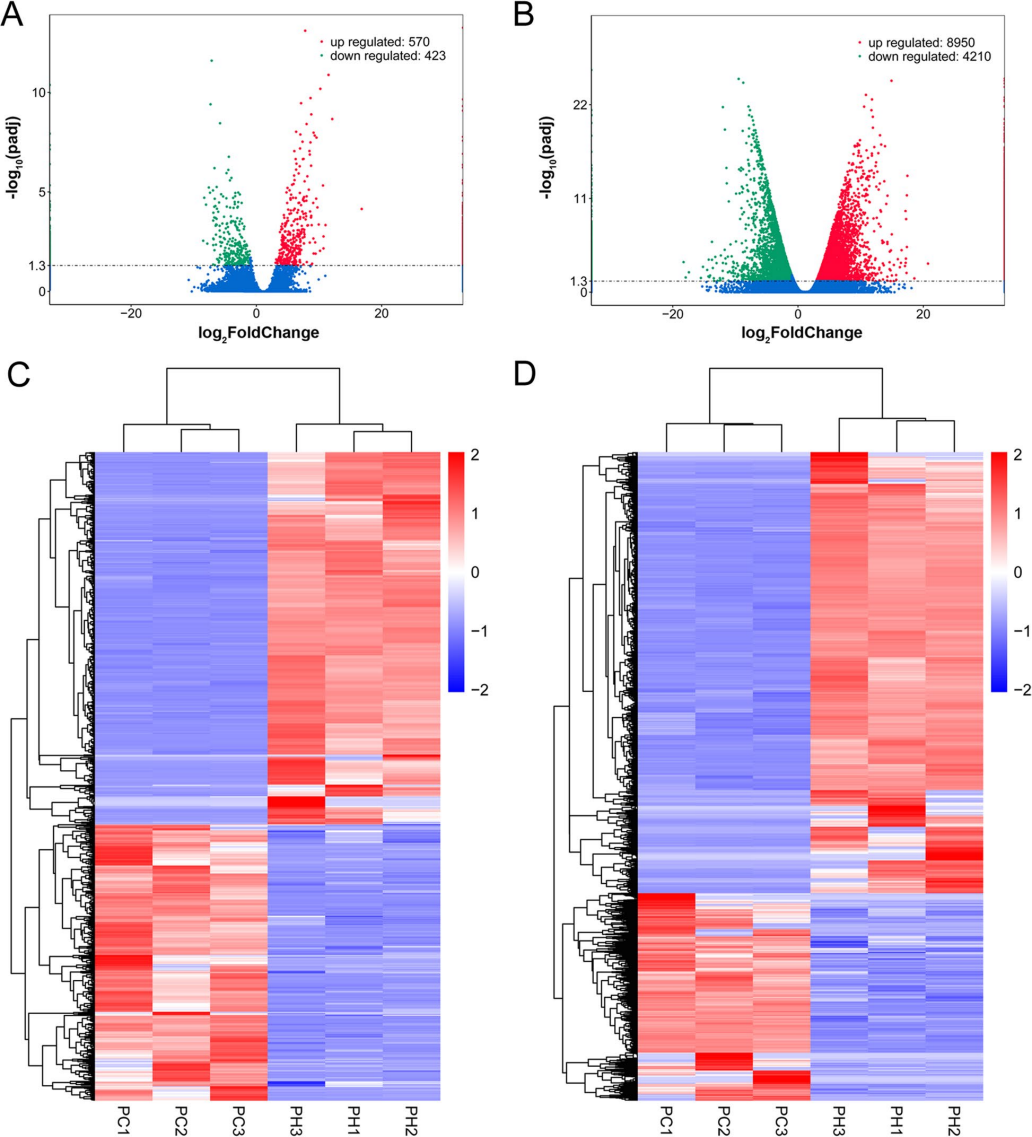
Identification of heat-responsive lncRNAs and mRNAs. **A** Volcano plot of the significantly differentially expressed lncRNAs; **B** Volcano plot of the significantly differentially expressed mRNAs; **C** Heatmap of the significantly differentially expressed lncRNAs; **D** Heatmap of the significantly differentially expressed mRNAs. Each heatmap was generated after transformation of the FPKM values into log_10_(FPKM + 1) values

**Fig. 6:**
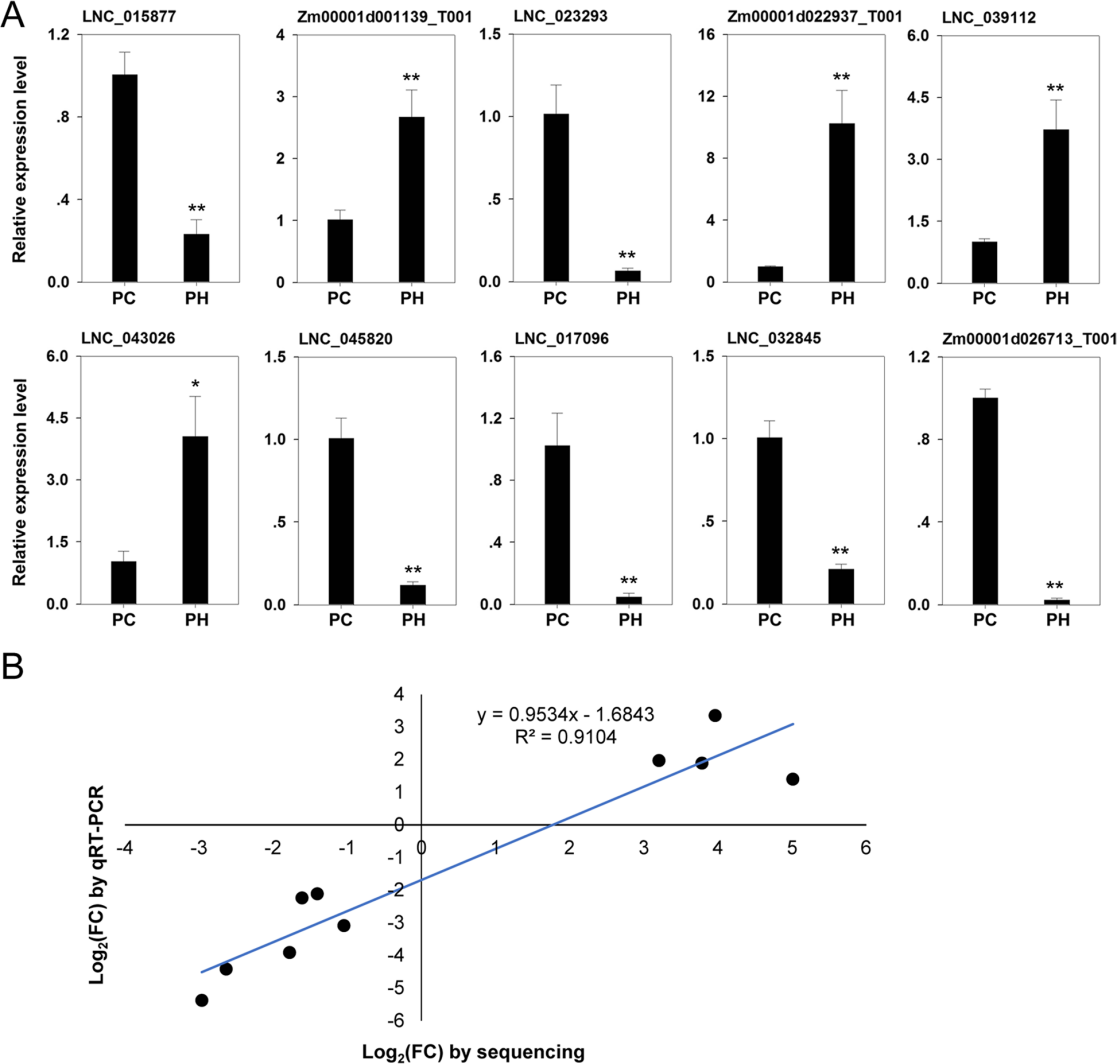
Expression verification of significantly differentially expressed lncRNAs using qRT-PCR analysis. **A.** Expression analysis of significantly differentially expressed lncRNAs; **B.** Correlation analysis of the sequencing data and qRT-PCR results

Expression pattern analysis was conducted to identify lncRNAs involved in the response to heat stress. By comparison of the control and heat-treatment groups, a total of 993 DElncRNAs were detected (Table S3), comprising 570 up-regulated members and 423 down-regulated members (Fig. 5A and C). Using the same screening criteria, 13,160 differentially expressed mRNA transcripts were identified, comprising 8,950 up-regulated transcripts and 4,210 down-regulated transcripts compared with the control group (Fig. 5B and D). We found that the number of the up-regulated genes exceeded that of the down-regulated members under heat stress. In particular, the difference in number of protein-coding genes between up- and down-regulated members was about two-fold. Heatmap analysis indicated that DElncRNAs or differentially expressed mRNA transcripts from the three biological replicates of each group were clustered, further indicating the strong correlation of the replicate samples. Ten DElncRNAs were chosen for qRT-PCR analysis to verify the expression patterns of the sequencing data. The expression pattern of the selected lncRNAs in the qRT-PCR experiments were largely consistent with those of the sequencing data (correlation coefficient 0.9104), which supported the reliability of the sequencing data (Fig. 6).

### Identification of target genes of DElncRNAs

Previous studies have demonstrated that the expression of target genes is regulated by lncRNAs in *cis*- and *trans*-regulatory approach [1]. Therefore, the target genes were identified to explore the potential biological roles of the detected DElncRNAs. With regard to *cis*-regulation, a total of 3,728 target genes were identified for the 993 DElncRNAs on the basis of their genomic location, of which 956 target genes showed significantly differential expression under heat stress treatment. A total of 24,038 *trans*-regulated target genes were identified for the 993 DElncRNAs on the basis of the correlation in their expression. Expression analysis indicated that 8,927 *trans*-regulated targets exhibited significantly differential expression between the control and heat stress groups. To further identify the potential key target genes in response to heat stress, the target genes common to *cis*- and *trans*-regulatory relationships were screened. A total of 953 common target genes were obtained and used for further analysis. The result indicated that the expression of most *cis*-regulated target genes was significantly correlated with the DElncRNAs, which further suggested the importance of these common targets.

### GO and KEGG analysis of target genes

**Fig. 7:**
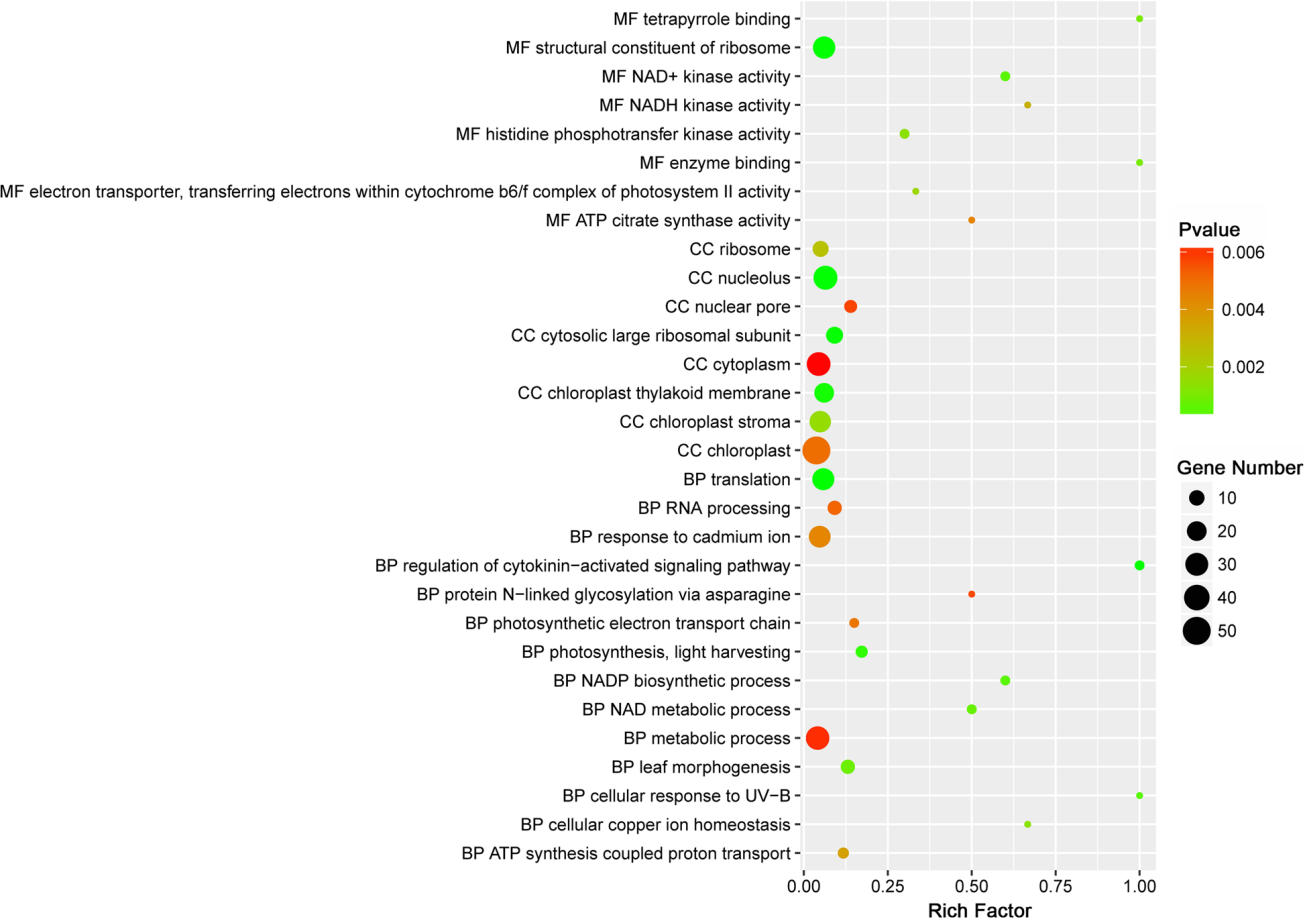
GO analysis of 953 common target genes of significantly differentially expressed lncRNAs

Analysis of GO and KEGG enrichment among the 953 common targets was performed to investigate their potential functional roles in addition to the associated DElncRNAs. GO classification revealed that 139 terms, comprising 24 CC terms, 56 BP terms, and 59 MF terms, were significantly enriched (*p*-value < 0.05) for the common targets. The 30 highly significant terms are indicated in Fig. 7. We found that the terms of “chloroplast” (GO:0009507), “cytoplasm” (GO:0005737) and “nucleolus” (GO:0005730) were most significantly annotated in the CC category. With regard to the BP category, the target genes were mainly enriched in the terms of “translation” (GO:0006412), “response to cadmium ion” (GO:0046686), “metabolic process” (GO:0008152), “photosynthesis, light harvesting” (GO:0009765), and “leaf morphogenesis” (GO:0009965). In addition, some target genes were enriched in the GO terms “response to reactive oxygen species” (GO:0000302), “response to stress” (GO:0006950), “response to water” (GO:0009415), “auxin-activated signaling pathway” (GO:0009734), and “regulation of cytokinin-activated signaling pathway” (GO:0080036), which may indicate the crucial roles of the enriched genes in the heat stress response (Table S4). In addition, the “structural constituent of ribosome” (GO:0003735), “NAD + kinase activity” (GO:0003951), and “histidine phosphotransfer kinase activity” (GO:0009927) terms were the most highly enriched in the MF category.

**Fig. 8:**
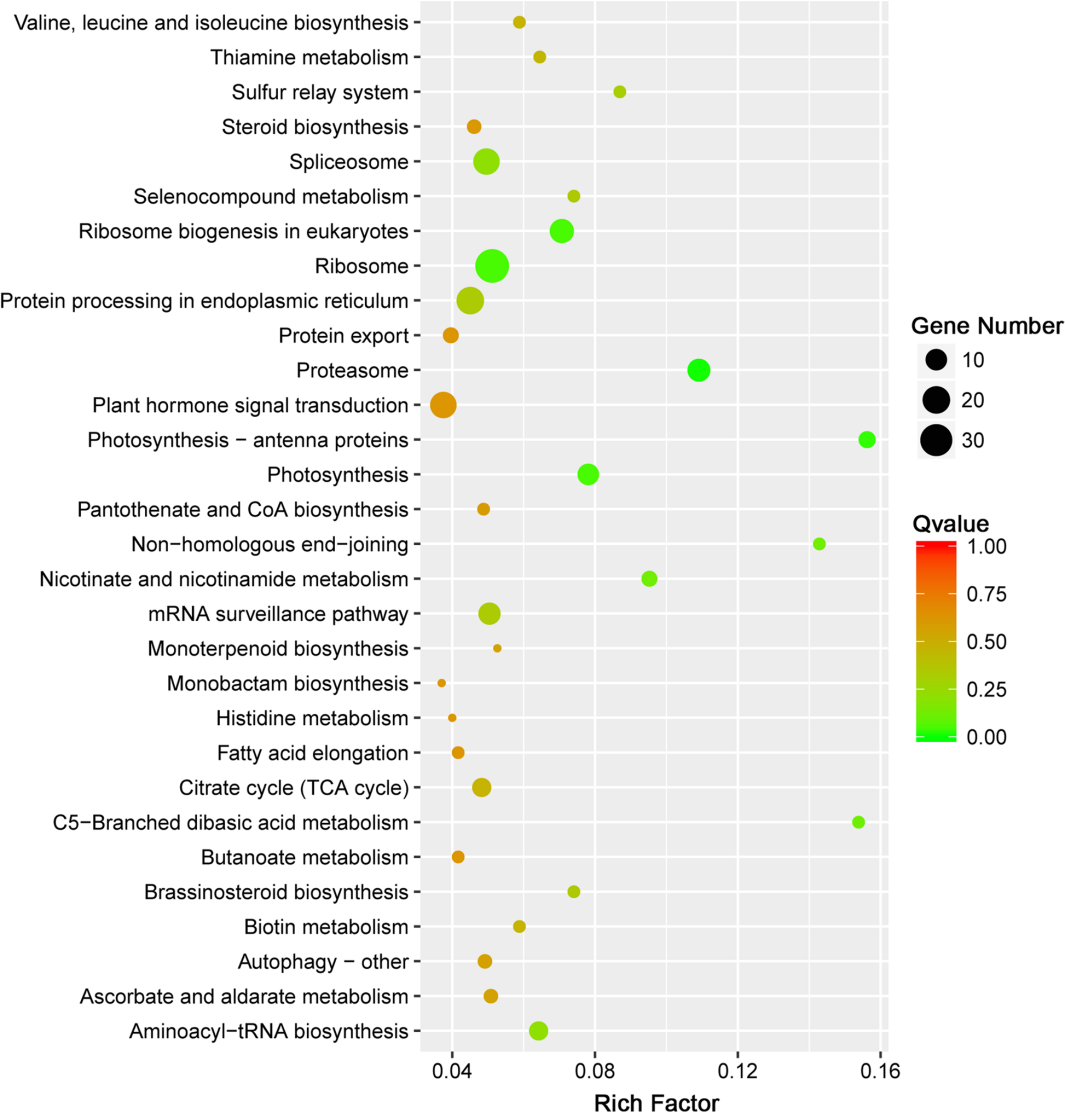
KEGG pathway analysis of 953 common target genes of significantly differentially expressed lncRNAs

The KEGG analysis revealed that the 953 target genes were annotated with 98 pathways. We found that a total of 73 target genes were significantly annotated (*q*-value < 0.05) with five pathways, comprising “proteasome” (ko03050), “ribosome” (ko03010), “ribosome biogenesis in eukaryotes” (ko03008), “photosynthesis-antenna proteins” (ko00196), and “photosynthesis” (ko00195). Meanwhile, pathways that may be associated with the heat response were also enriched for some target genes. For example, 18 genes were enriched in the “spliceosome” (ko03040) and “plant hormone signal transduction” (ko04075) pathways, and six genes were enriched in the “MAPK signaling pathway-plant” (ko04016) (Fig. 8).

### LncRNA mediated regulatory network in response to heat stress

**Fig. 9:**
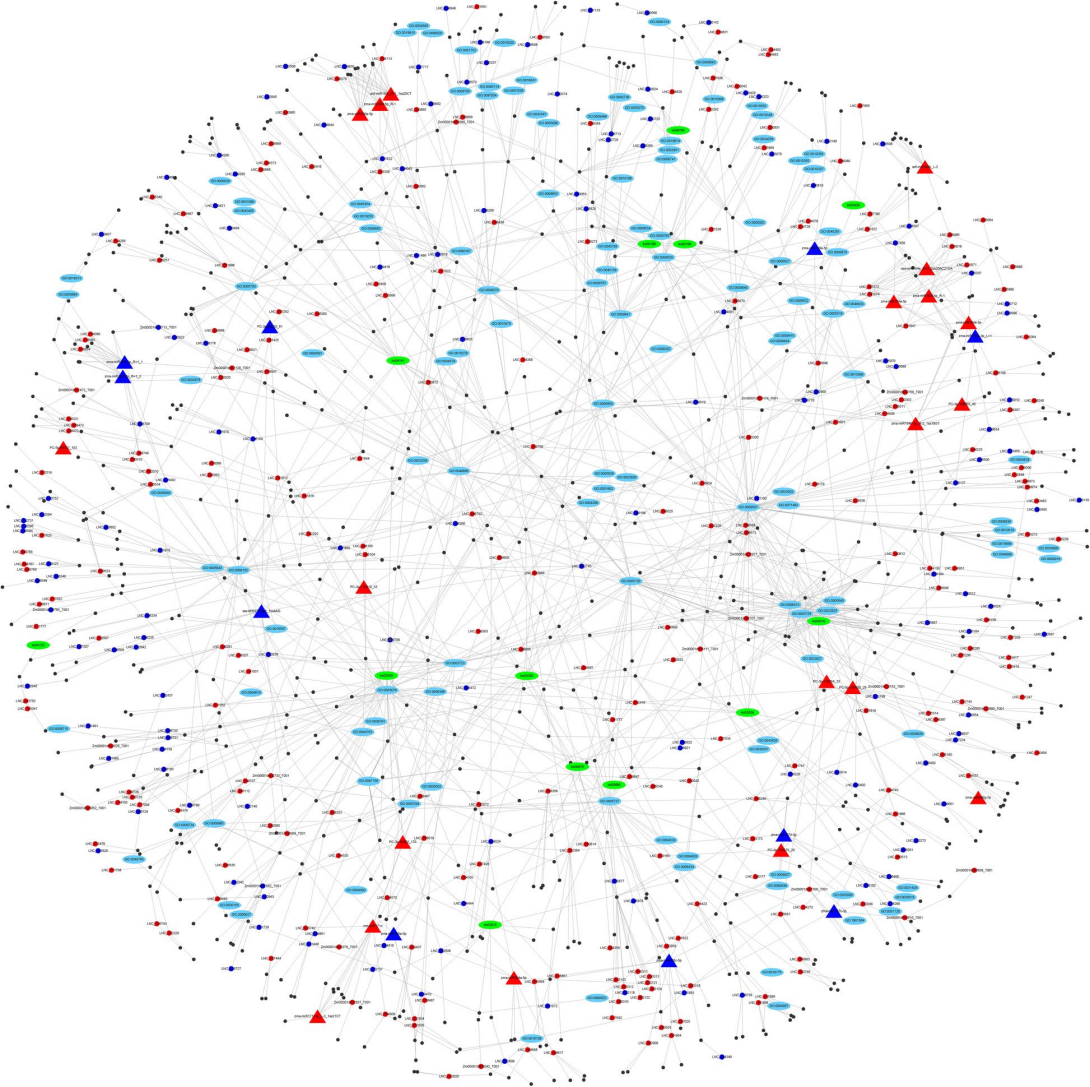
LncRNA-mediated regulatory network in the response to heat stress in the maize inbred line CM1. Triangles represent significantly differentially expressed miRNAs; Colored dots (red and green) represent significantly differentially expressed lncRNAs; Up-regulated expression is indicated by red, down-regulated expression is indicated by green; Black dots represent target genes of significantly differentially expressed lncRNAs and miRNAs; Blue and green ellipses represent significantly enriched GO terms and KEGG pathways (*p*-value < 0.05) for the target genes, respectively

During the process of plant evolution, a complex regulatory mechanism has evolved to cope with the adverse effects of abiotic stresses. Increasing evidence indicates that all protein-coding genes, miRNAs and lncRNAs have important biological functions in various abiotic stress responses. In our previous study, 35 DEMs were identified in CM1 under heat stress and their target mRNAs were predicted using degradome sequencing [38]. The regulatory relationships of these 35 DEMs and the detected DElncRNAs were further predicted in the present study. We constructed a lncRNA-miRNA-mRNA regulatory network based on the results of high-throughput sequencing of CM1 in response to heat stress, and include the enriched GO terms and KEGG pathways for the targets of DElncRNAs (Fig. 9). The network provided clues for discovery of the crucial key heat-responsive lncRNAs and their target genes. For example, 11 genes enriched in the “response to stress” (GO:0006950) GO term, were detected as the targets of 12 DElncRNAs. We noted that two of the 12 DElncRNAs were targeted by six DEMs (Tables S5 and S6). In particular, two heat shock proteins (Zm00001d028555 and Zm00001d052194) and one late embryogenesis abundant protein (Zm00001d040659) were detected among the 11 targets. Eighteen target genes enriched in the “spliceosome” (ko03040) pathway, were regulated by 20 DElncRNAs, and three DElncRNAs were targeted by three DEMs. Therefore, identification of crucial target genes involved in the heat stress response, and further exploration of crucial lncRNA-miRNA-mRNA molecular regulatory modules through the regulatory network, is important for molecular breeding to cultivate the heat-tolerant maize germplasm.

## Discussion

Through long-term adaptive evolution, plants have developed sophisticated and complex response mechanisms to cope with the adverse effects of heat stress [40, 41]. In response to high temperature during the growth and development cycle of plants, thousands of transcripts, including protein-coding genes and non-coding RNAs (e.g., miRNAs and lncRNAs), are significantly differentially regulated. In particular, many genes involved in stress responses have been identified using a variety of methods and techniques, and significantly improved tolerance of plants to heat stress has been achieved by applying these genes in molecular breeding. Therefore, to understand plants' signal perception and the molecular regulatory mechanisms of the heat stress response, is important for cultivating new heat-tolerant germplasm in plants.

As important regulatory molecules that modulate gene expression, lncRNAs are involved in diverse biological processes in eukaryotes [4, 42]. Moreover, a growing number of lncRNAs have been demonstrated for their important regulatory functions in abiotic stress responses, such as drought and high temperature, and stress-responsive lncRNAs have been systematically screened in multiple species using high-throughput sequencing technology [19–22, 43–45]. Our previous study demonstrated the strong resistance of the maize elite inbred line CM1 to heat stress and its important genetic contribution to heat resistant maize varieties [27]. In the present study, systematic identification and analysis of lncRNAs was performed to further explore the molecular mechanism of lncRNAs in the response to heat stress in this inbred line. In total, 53,249 lncRNAs, comprising 259 known lncRNAs and 52,990 novel lncRNAs, were identified in CM1 under the non-stress and heat stress conditions. Among the identified lncRNAs, the lincRNA type accounted for the majority (95.56%) followed by intronic lncRNA (2.47%). Comparative analysis of the expression patterns and structural characteristics of the detected lncRNAs and mRNAs revealed significant differences in their expression levels, exon number, and transcript length. The overall abundance of expressed lncRNAs was lower than that of mRNAs, the transcript length was generally shorter, and most lncRNAs contained one or two exons, which were similar to those reported in previous studies [10, 46]. Under the heat stress treatment, 993 DElncRNAs were identified, comprising 570 up-regulated and 423 down-regulated members. These findings suggested that the expression of many lncRNAs may be induced by heat stress, which also indicated the crucial regulatory roles of lncRNAs in heat stress response.

Although lncRNAs lack protein-coding ability, they can execute their biological functions through regulating the expression of target genes by means of a *cis*- or *trans*-mediated mechanism [1, 42]. Thus, identification of lncRNA target genes is essential for understanding how lncRNAs perform their biological functions. A total of 3,728 *cis*-regulated and 24,038 *trans*-regulated target genes were identified for the 993 DElncRNAs. To identify the crucial target genes involved in the response to heat stress, 953 target genes common to *cis*- and *trans*-regulatory lncRNAs that showed significantly differential expression under heat stress treatment were detected. Enrichment analysis indicated that these common target genes were significantly enriched with 139 GO terms and annotated with five significantly enriched KEGG pathways. We noted that the enrichment results were partly similar to the enriched GO terms and KEGG pathways for differentially expressed genes and lncRNA target genes in response to heat stress reported in previous studies [27, 47, 48], further suggesting that the lncRNA targets may perform essential functional roles in the regulation of the heat response.

**Fig. 10:**
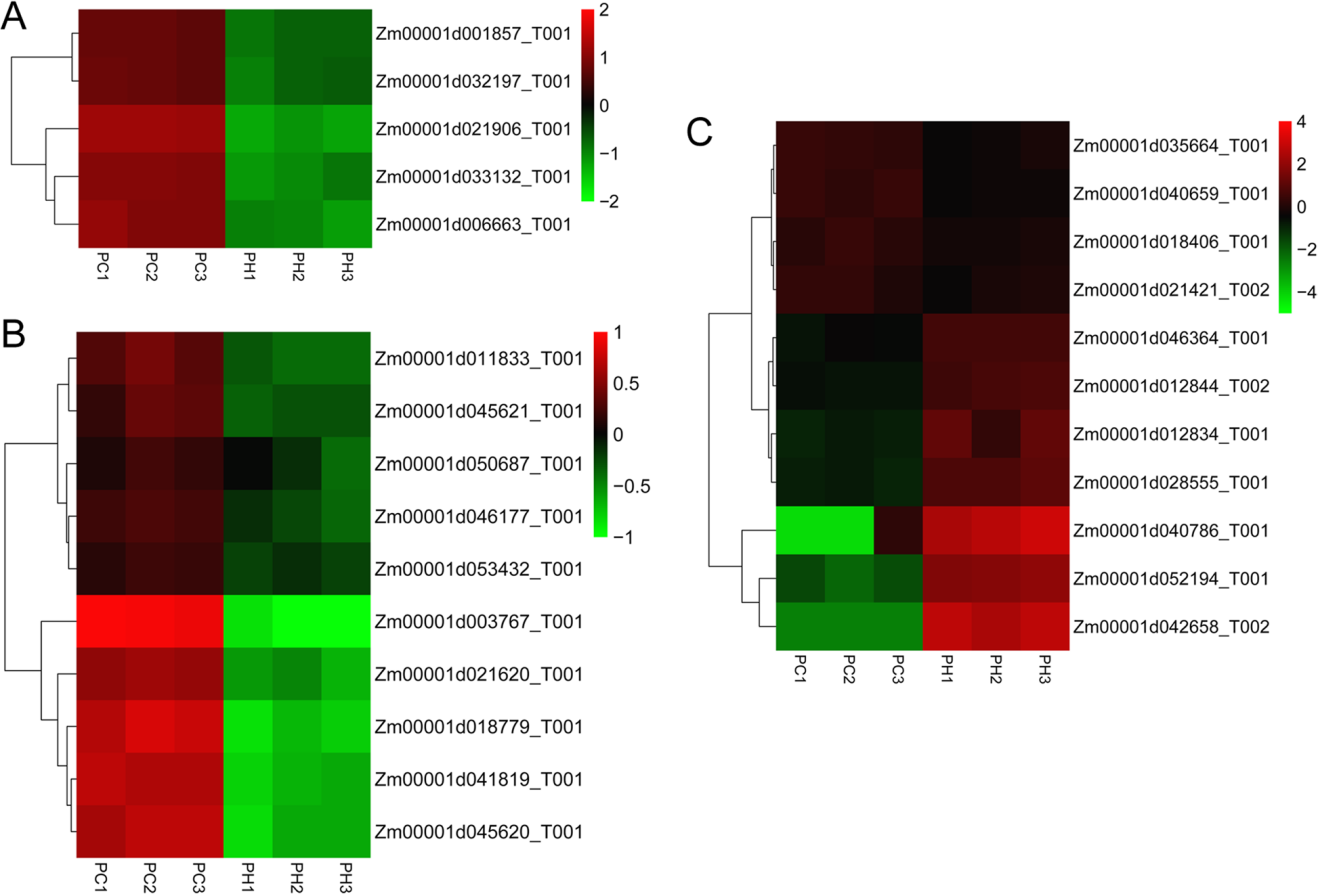
Expression analysis of photosynthesis-related and stress-response genes annotated with GO and KEGG. **A** Heatmap of the genes enriched in the “photosynthesis-antenna proteins” pathway; **B** Heatmap of the genes enriched in the “photosynthesis” pathway; **C** Heatmap of the genes enriched in the “response to stress” biological process term

When plants are subjected to heat stress, photosynthesis can be significantly inhibited because of the disruption of the structural organization of chloroplasts and reduction in photosystem II activity, thereby adversely affecting crop growth and production [41]. We observed that five and three lncRNA targets were enriched in the “photosynthesis, light harvesting” and “photosynthetic electron transport chain” GO terms, respectively. In particular, five and ten target genes were annotated with the “photosynthesis-antenna proteins” and “photosynthesis” KEGG pathways, respectively (Figs S1 and S2). Accordingly, these genes were significantly down-regulated under heat stress (Fig. 10A and B) and the majority showed large fold changes in expression. These results were consistent with previous findings [27, 49], which may indicate that a correction between gene expression changes and inhibition of photosynthesis occurs under heat stress. Previous studies indicate that oxidative stress and osmotic pressure changes can be caused by heat stress, which can lead to accumulation of reactive oxygen species and drought stress, respectively [41, 50]. We noted that two and eleven genes were assigned to the GO terms “response to reactive oxygen species” and “response to stress”, respectively. It was notable that genes enriched in the “response to stress” included two heat shock proteins (up-regulated with large fold changes) and one late embryogenesis abundant protein (down-regulated) (Fig. 10C).

Environmental stresses, such as high temperature, can also affect the production of hormones. For example, the expression of certain auxin synthesis genes may be regulated by high temperature [50]. In the BP category, six genes were annotated with the “auxin-activated signaling pathway”, and 18 genes were annotated with the “plant hormone signal transduction” pathway. In addition, some genes were annotated with the “MAPK signaling pathway-plant” (6 genes) and “spliceosome” (18 genes) pathways, which have been shown to play essential roles in many biological processes as well as stress response [50, 51]. These findings indicated the important biological functions of these target genes in the regulation of the heat stress response.

Taken together, the present results revealed that the mechanisms of the plant response to heat stress included a serious of complex physiological and molecular responses, thus forming a regulatory network composed of multiple genes (e.g., mRNAs, lncRNAs, and miRNAs) and signaling pathways. Based on previous research, the regulatory relationships of 993 DElncRNAs and 35 DEMs, was further predicted in the present study. Thus, a lncRNA-mediated regulatory network, including DElncRNAs, DEMs, their target genes, and the enrichment annotations, was constructed using Cytoscape software. The regulatory network lays an important foundation for further understanding the genetic regulatory mechanism in response to heat stress in the maize inbred line CM1, and provides important clues for identification of important heat-responsive genes in maize.

## Conclusion

Systematic identification of lncRNAs was performed in the maize inbred line CM1 under heat stress using high-throughput sequencing. Compared with mRNAs, the detected lncRNAs exhibited significant differences in their expression profiles and structural characteristics. A total of 993 DElncRNAs were identified, and 953 target genes common to *cis*- and *trans*-regulatory of DElncRNAs were differentially expressed between the control and heat stress treatments. Enrichment analysis revealed that the target genes were annotated with a number of important biological processes and pathways, such as photosynthesis and plant hormone signal transduction. A lncRNA-mediated regulatory network in response to heat stress was constructed based on the detected heat-responsive lncRNAs, miRNAs, target genes, and their functional annotations, which is of great significance for the cultivation of heat-tolerant germplasm through maize molecular breeding.

## Supplementary Information

**Additional file 1: Figure S1.** KEGG enrichment map of the photosynthesis-antenna proteins pathway.

**Additional file 2: Figure S2.** KEGG enrichment map of the photosynthesis pathway.

**Additional file 3: Table S1.** Primers of selected lncRNAs used for qRT-PCR analysis.

**Additional file 4: Table S2.** Statistics of lncRNA sequencing from the six libraries.

**Additional file 5: Table S3.** Detailed information of the 993 significantly differentially expressed lncRNAs.

**Additional file 6: Table S4.** GO terms enriched in the biological process category for the 953 common targets.

**Additional file 7: Table S5.** Regulatory relationships of DElncRNAs and the 953 common targets.

**Additional file 8: ** Table S6. Regulatory relationships of the DEMs and DElncRNAs predicted by psRNATarget.

## Data Availability

The raw data of the six libraries were deposited in the Sequence Read Archive (SRA) database of the NCBI under the accession no. PRJNA792891.
