# Supplementary figures and images for: Identification and characterization of heat-responsive lncRNAs in maize inbred line CM1
Source: BMC Genomics. 2022 Mar 16;23:208. doi: 10.1186/s12864-022-08448-1 (PMC8925227; doi:10.1186/s12864-022-08448-1)

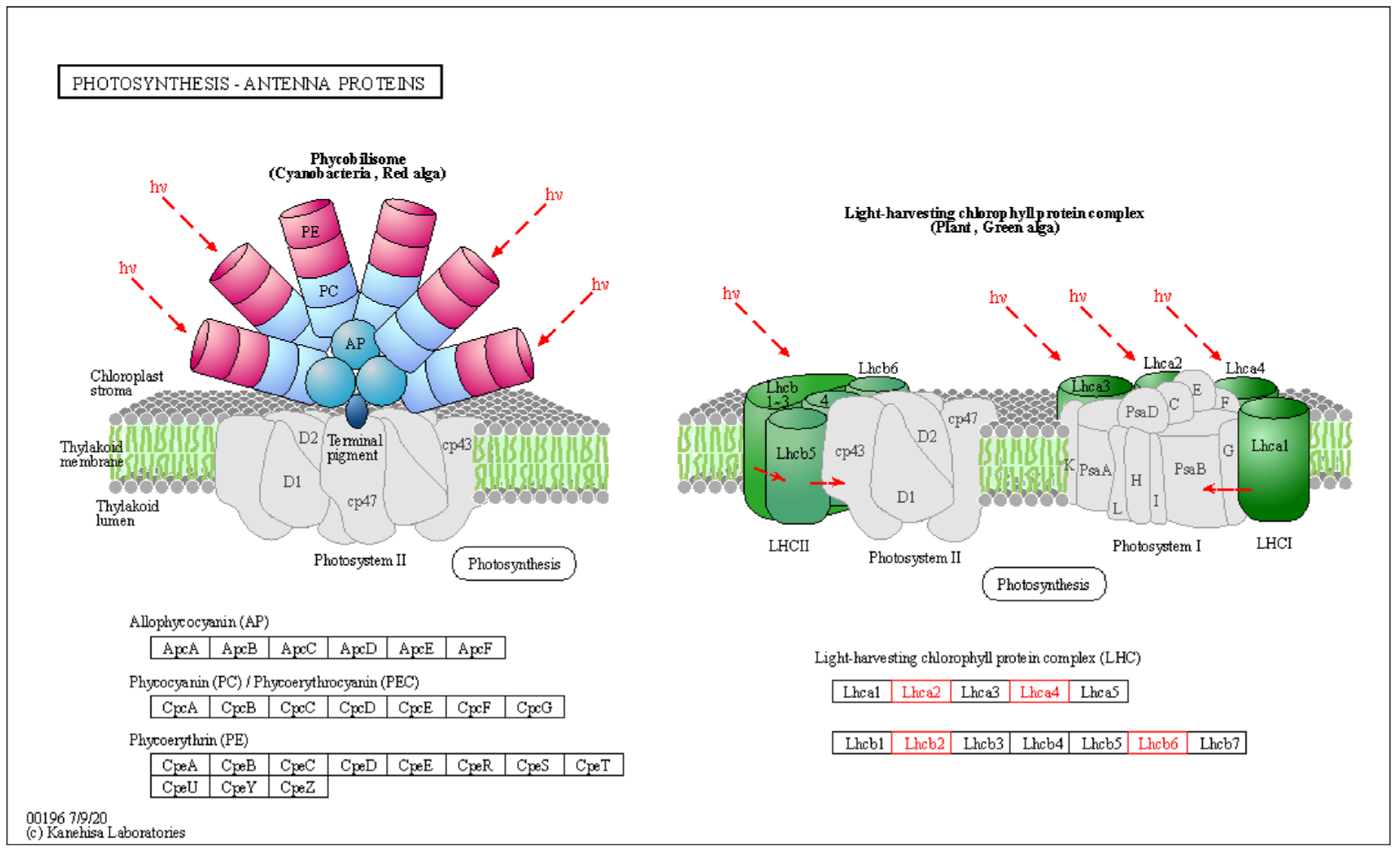

Supplement: Supplementary file 1 — Additional file 1: Figure S1. KEGG enrichment map of the photosynthesis-antenna proteins pathway. [file 12864_2022_8448_MOESM1_ESM.tif]

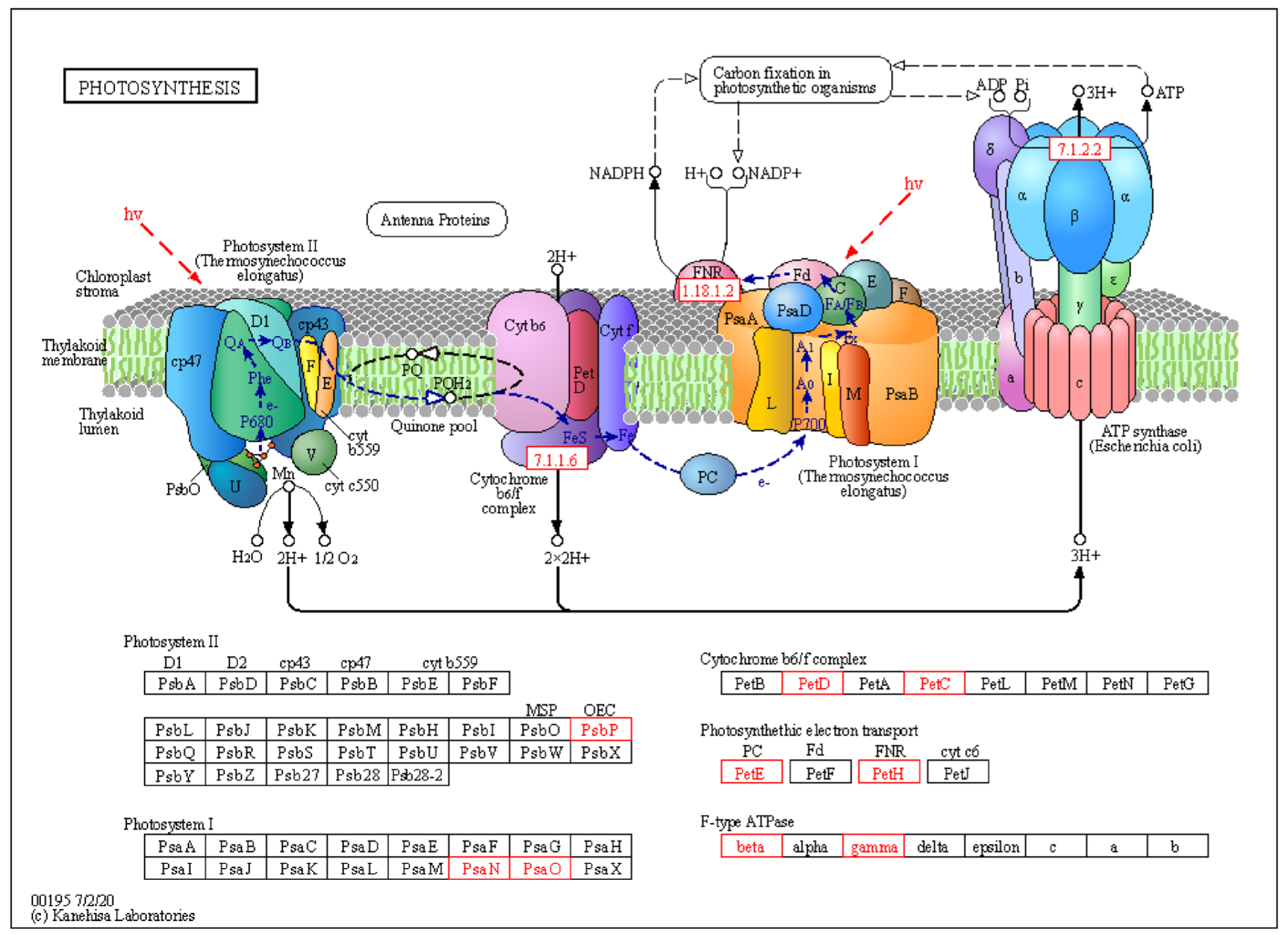

Supplement: Supplementary file 2 — Additional file 2: Figure S2. KEGG enrichment map of the photosynthesis pathway. [file 12864_2022_8448_MOESM2_ESM.tif]
