# Supplementary material for: Identification and characterization of heat-responsive lncRNAs in maize inbred line CM1
Source: BMC Genomics. 2022 Mar 16;23:208. doi: 10.1186/s12864-022-08448-1 (PMC8925227; doi:10.1186/s12864-022-08448-1)
Supplement: Supplementary file 4 — Additional file 4: Table S2. Statistics of lncRNA sequencing from the six libraries. [file 12864_2022_8448_MOESM4_ESM.docx]

**Table S2. Statistics of lncRNA sequencing from the six libraries.**

| **Samples** | **Raw reads** | **Clean reads** | **Q20 (%)** | **Q30 (%)** | **GC (%)** | **Total mapped (%)** |
| --- | --- | --- | --- | --- | --- | --- |
| PC1 | 100049182 | 99733092 | 96.81 | 91.36 | 46.08 | 93.56 |
| PC2 | 117573496 | 117230874 | 97.22 | 92.22 | 46.4 | 94.41 |
| PC3 | 96258000 | 95964646 | 97.06 | 91.90 | 46.66 | 93.24 |
| PH1 | 142765552 | 142315414 | 97.07 | 91.92 | 47.94 | 89.82 |
| PH2 | 120032270 | 119610414 | 97.03 | 91.91 | 48.77 | 89.21 |
| PH3 | 110898036 | 110519750 | 97.00 | 91.89 | 50.40 | 81.17 |

PC, seedlings under control condition; PH, seedlings under heat treatment; 1-3, three biological replicates.
