# Supplementary material for: Identification and characterization of heat-responsive lncRNAs in maize inbred line CM1
Source: BMC Genomics. 2022 Mar 16;23:208. doi: 10.1186/s12864-022-08448-1 (PMC8925227; doi:10.1186/s12864-022-08448-1)
Supplement: Supplementary file 6 — Additional file 6: Table S4. GO terms enriched in the biological process category for the 953 common targets. [file 12864_2022_8448_MOESM6_ESM.docx]

**Table S4. GO terms enriched in the biological process category for the 953 common targets.**

| **Category** | **Term** | **Ontology** | **Gene number** | **p-value** |
| --- | --- | --- | --- | --- |
| GO:0006412 | translation | BP | 27 | 1.8E-07 |
| GO:0080036 | regulation of cytokinin-activated signaling pathway | BP | 3 | 4.93E-06 |
| GO:0009765 | photosynthesis, light harvesting | BP | 5 | 0.000141 |
| GO:0006741 | NADP biosynthetic process | BP | 3 | 0.000473 |
| GO:0071493 | cellular response to UV-B | BP | 2 | 0.000474 |
| GO:0019674 | NAD metabolic process | BP | 3 | 0.00064 |
| GO:0009965 | leaf morphogenesis | BP | 8 | 0.000716 |
| GO:0006878 | cellular copper ion homeostasis | BP | 2 | 0.001241 |
| GO:0015986 | ATP synthesis coupled proton transport | BP | 4 | 0.003612 |
| GO:0046686 | response to cadmium ion | BP | 26 | 0.004415 |
| GO:0009767 | photosynthetic electron transport chain | BP | 3 | 0.004806 |
| GO:0006396 | RNA processing | BP | 8 | 0.005249 |
| GO:0018279 | protein N-linked glycosylation via asparagine | BP | 2 | 0.005731 |
| GO:0008152 | metabolic process | BP | 32 | 0.006129 |
| GO:0000302 | response to reactive oxygen species | BP | 2 | 0.007514 |
| GO:0006950 | response to stress | BP | 11 | 0.007711 |
| GO:0032502 | developmental process | BP | 2 | 0.008527 |
| GO:0006662 | glycerol ether metabolic process | BP | 5 | 0.009721 |
| GO:0010019 | chloroplast-nucleus signaling pathway | BP | 2 | 0.011316 |
| GO:0006434 | seryl-tRNA aminoacylation | BP | 2 | 0.012162 |
| GO:0006827 | high-affinity iron ion transmembrane transport | BP | 1 | 0.016369 |
| GO:0043547 | positive regulation of GTPase activity | BP | 2 | 0.016653 |
| GO:0051603 | proteolysis involved in cellular protein catabolic process | BP | 3 | 0.017823 |
| GO:0055086 | nucleobase-containing small molecule metabolic process | BP | 1 | 0.01913 |
| GO:0055070 | copper ion homeostasis | BP | 1 | 0.019458 |
| GO:0044763 | single-organism cellular process | BP | 5 | 0.020329 |
| GO:0010196 | nonphotochemical quenching | BP | 2 | 0.020442 |
| GO:0000918 | barrier septum site selection | BP | 1 | 0.021232 |
| GO:0008643 | carbohydrate transport | BP | 4 | 0.021446 |
| GO:0006302 | double-strand break repair | BP | 2 | 0.023001 |
| GO:0045454 | cell redox homeostasis | BP | 8 | 0.023261 |
| GO:0006005 | L-fucose biosynthetic process | BP | 1 | 0.023719 |
| GO:0042350 | GDP-L-fucose biosynthetic process | BP | 1 | 0.023719 |
| GO:0006664 | glycolipid metabolic process | BP | 1 | 0.027467 |
| GO:0009415 | response to water | BP | 1 | 0.027467 |
| GO:0009626 | plant-type hypersensitive response | BP | 3 | 0.031002 |
| GO:0015996 | chlorophyll catabolic process | BP | 2 | 0.033126 |
| GO:0019915 | lipid storage | BP | 1 | 0.036944 |
| GO:0080020 | regulation of coenzyme A biosynthetic process | BP | 1 | 0.036944 |
| GO:0031120 | snRNA pseudouridine synthesis | BP | 1 | 0.037102 |
| GO:0000038 | very long-chain fatty acid metabolic process | BP | 2 | 0.037268 |
| GO:0009791 | post-embryonic development | BP | 4 | 0.040589 |
| GO:0009853 | photorespiration | BP | 2 | 0.041557 |
| GO:0032259 | methylation | BP | 12 | 0.041941 |
| GO:0009734 | auxin-activated signaling pathway | BP | 6 | 0.043657 |
| GO:0010478 | chlororespiration | BP | 1 | 0.043943 |
| GO:0080167 | response to karrikin | BP | 7 | 0.044104 |
| GO:1901564 | organonitrogen compound metabolic process | BP | 1 | 0.044313 |
| GO:0030155 | regulation of cell adhesion | BP | 1 | 0.044359 |
| GO:2000027 | regulation of organ morphogenesis | BP | 1 | 0.044359 |
| GO:0050794 | regulation of cellular process | BP | 4 | 0.045254 |
| GO:0048765 | root hair cell differentiation | BP | 2 | 0.045432 |
| GO:0010352 | lithium ion export | BP | 1 | 0.045491 |
| GO:0080114 | positive regulation of glycine hydroxymethyltransferase activity | BP | 1 | 0.048395 |
| GO:0097054 | L-glutamate biosynthetic process | BP | 1 | 0.048395 |
| GO:0050954 | sensory perception of mechanical stimulus | BP | 1 | 0.04985 |
